# Methylphenidate and galantamine in patients with vascular cognitive impairment–the proof-of-principle study STREAM-VCI

**DOI:** 10.1186/s13195-019-0567-z

**Published:** 2020-01-07

**Authors:** Jolien F. Leijenaar, Geert Jan Groeneveld, Erica S. Klaassen, Anna E. Leeuwis, Philip Scheltens, Henry C. Weinstein, Joop M. A. van Gerven, Frederik Barkhof, Wiesje M. van der Flier, Niels D. Prins

**Affiliations:** 1 Alzheimer Center Amsterdam, Department of Neurology, Amsterdam Neuroscience, VU University Medical Center, Amsterdam UMC, P.O. Box 7057, 1007 MB Amsterdam, The Netherlands; 2 Centre for Human Drug Research, Leiden, The Netherlands; 3 Department of Neurology, Onze Lieve Vrouwe Gasthuis (OLVG) West, Amsterdam, The Netherlands; 4 Department of Radiology and Nuclear Medicine, Amsterdam Neuroscience, VU University Medical Center, Amsterdam UMC, Amsterdam, The Netherlands; 5 Institutes of Neurology and Healthcare Engineering, UCL, London, UK; 6 Department of Epidemiology, Amsterdam Neuroscience, VU University Medical Center, Amsterdam UMC, Amsterdam, The Netherlands; 7 Brain Research Center, Amsterdam, The Netherlands

**Keywords:** Vascular cognitive impairment, Methylphenidate, Galantamine, Cognition, Vascular dementia, MCI

## Abstract

**Background:**

To date, no symptomatic treatment is available for patients with vascular cognitive impairment (VCI). In the proof-of-principle study Symptomatic Treatment of Vascular Cognitive Impairment (STREAM-VCI), we investigated whether a single dose of a monoaminergic drug (methylphenidate) improves executive functioning and whether a single dose of a cholinergic drug (galantamine) improves memory in VCI patients.

**Methods:**

STREAM-VCI is a single-center, double-blind, three-way crossover trial. We included 30 VCI patients (Mini-Mental State Examination (MMSE) ≥ 16 and Clinical Dementia Rating score 0.5–1.0) with cerebrovascular pathology on MRI. All patients received single doses of methylphenidate (10 mg), galantamine (16 mg), and placebo in random order on three separate study visits. We used the NeuroCart®, a computerized test battery, to assess drug-sensitive cognitive effects. Predefined main outcomes, measured directly after a single dose of a study drug, were (i) change in performance on the adaptive tracker for executive functioning and (ii) performance on the Visual Verbal Learning Test-15 (VVLT-15) for memory, compared to placebo. We performed mixed model analysis of variance.

**Results:**

The study population had a mean age of 67 ± 8 years and MMSE 26 ± 3, and 9 (30%) were female. Methylphenidate improved performance on the adaptive tracker more than placebo (mean difference 1.40%; 95% confidence interval [CI] 0.56–2.25; *p* = 0.002). In addition, methylphenidate led to better memory performance on the VVLT-15 compared to placebo (mean difference in recalled words 0.59; 95% CI 0.03–1.15; *p* = 0.04). Galantamine did not improve performance on the adaptive tracker and led to worse performance on delayed recall of the VVLT-15 (mean difference − 0.84; 95% CI − 1.65, − 0.03; *p* = 0.04). Methylphenidate was well tolerated while galantamine produced gastrointestinal side effects in a considerable number of patients.

**Conclusions:**

In this proof-of-principle study, methylphenidate is well tolerated and improves executive functioning and immediate recall in patients with VCI. Galantamine did not improve memory or executive dysfunction. Results might be influenced by the considerable amount of side effects seen.

**Trial registration:**

http://www.clinicaltrials.gov. Registration number: NCT02098824. Registration date: March 28, 2014.

## Background

Vascular cognitive impairment (VCI) is an important cause of cognitive decline and dementia [1]. Patients with VCI most often show executive dysfunction and/or memory impairment as most prominent cognitive symptoms [2]. However, the presence and extent of these symptoms varies greatly between patients. Currently, there is no approved treatment for patients with VCI that can reduce cognitive symptoms.

Executive functioning is largely related to the monoaminergic neurotransmitter system and memory largely to the cholinergic neurotransmitter system [3, 4]. Localized vascular brain injury may cause damage to one of these neurotransmitter systems, causing either monoaminergic or cholinergic deficits [5]. These deficits might in turn cause executive dysfunction or memory impairment in VCI, depending on the affected neurotransmitter system. This raises the possibility that the failing neurotransmitter systems can be supported with monoamine or cholinergic agonists.

Methylphenidate may improve executive functioning by increasing norepinephrine and dopamine concentrations in the synaptic cleft [6]. While some studies in patients with dementia due to Alzheimer’s disease (AD) have suggested an effect of methylphenidate on global cognition and on attention [7–10], other studies have not consistently supported the results [11]. In patients with VCI, only 1 small older open label longitudinal study in 15 patients with dementia found that methylphenidate slightly improved scores on the Mini-Mental State Examination (MMSE) [12]. Galantamine increases the availability of acetylcholine in the synaptic cleft and is an allosteric modulator of nicotinic acetylcholine receptors [13]. Although galantamine is an approved drug for dementia due to AD, the effect of galantamine in VCI remains uncertain [14, 15]. Nonetheless, several studies have suggested a small cognitive benefit of galantamine over placebo in patients with VCI [16, 17]. This indicates that although VCI is a chronically progressive condition with irreparable damage of white and gray matter, executive dysfunction and memory impairment due to partially damaged neuronal connections may still respond to acute pharmacological stimulation.

In the proof-of-principle study “Symptomatic Treatment of Vascular Cognitive Impairment” (STREAM-VCI), we hypothesized that methylphenidate may improve executive function and that galantamine may improve memory in patients with VCI.

## Methods

### Trial design

The STREAM-VCI was a single-center, double-blind, three-way, crossover study, among patients with VCI. All patients gave written informed consent. The protocol of this study was approved by the Medical Ethics Committee of Amsterdam UMC and the competent authority. The study was conducted according to the Dutch Act on Medical Research involving Human Subjects (WMO) and in compliance with good clinical practice (ICH-GCP).

### Patients

We included 30 patients with a diagnosis of VCI, according to the definitions of the American Heart Association/American Stroke Association [18]. The most important inclusion criteria were as follows: a clinical diagnosis of mild cognitive impairment (MCI) or dementia with imaging evidence of vascular brain injury (white matter hyperintensities (Fazekas ≥ 2), (lacunar) infarcts, or (micro) hemorrhages), an MMSE ≥ 16, and a Clinical Dementia Rating score 0.5–1.0. The main exclusion criteria were any contraindication for study medication and other causes that could explain cognitive symptoms. The presence of comorbid AD pathology was not an exclusion criterion. An extensive overview of the inclusion and exclusion criteria has been published previously [19].

### Procedures

The study consisted of a screening day and three study visits. On the screening day, patients were tested for eligibility through a full medical screening (including medical history and physical examination) and an MMSE. Presence of vascular risk factors was based on medical history and medication use. When eligibility was confirmed, patients received in randomized order single doses of galantamine, methylphenidate, and placebo on three separate study visits with a washout period of a week between each study visit. On the study visits, patients performed central nervous system (CNS) tests before and after administration of the study drug. Patients were enrolled between April 2014 and September 2017.

### Dosing rationale

We administered a single dose of 10 mg (two tablets of 5 mg) methylphenidate and a single dose of 16 mg (two tablets of 8 mg) galantamine. The dose of methylphenidate was chosen as it was found to be effective on apathy and safe in patients with dementia due to AD [11]. The dose of galantamine was chosen based on ongoing study clinical trials showing that a single dose of at least 16 mg is necessary to demonstrate pharmacodynamic effects, and has an acceptable side effect profile when the up-titration period is skipped (CHDR0915, not yet published). To ensure blinding, methylphenidate, galantamine, and placebo were overencapsulated and looked identical.

### Visual rating MRI

On the screening day, all patients underwent a brain MRI scan, including FLAIR, and T1-, T2-, and T2*-weighted images. All scans were reviewed by a neuroradiologist for unexpected gross abnormalities. White matter hyperintensities, microbleeds, lacunes, cortical infarcts, and atrophy scores were visually rated as previously described [19].

### Pharmacodynamic assessment

**Fig. 1:**
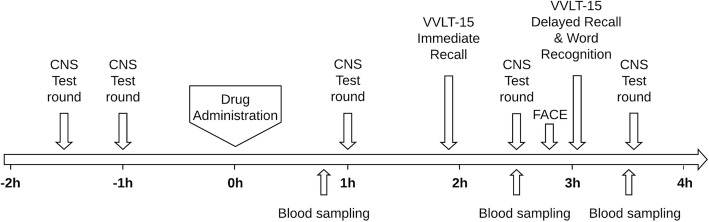
Overview of the activities on a study day. The arrows represent when a test round or test is started. CNS test round encompasses all tests with exception of the VVLT-15 and the FACE. At time point 0, the study medication was administered

The NeuroCart®, a computerized CNS test battery, is developed to study effects of CNS-active drugs on a wide range of central nervous system domains [20]. Pharmacodynamic assessment was performed using a previously described protocol [19]. In short, the following tests were used to measure executive functioning: adaptive tracking (a pursuit-tracking task), Stop Signal Task (an inhibition task), and N-back task (assessing working memory). Memory was assessed by the Visual Verbal Learning Test-15 (VVLT-15) and the Face Encoding Recognition Task (FACE). The VVLT-15 contains three different subtests. The immediate word recall test was performed first; after an interval of approximately 60 min, the delayed word recall test and subsequently the delayed word recognition test were performed. We also measured psychomotor speed using saccadic and smooth pursuit eye movements, and subjective drug effects using the Bond and Lader Visual Analog Scale (VAS) and a resting state eyes-closed pharmaco-electroencephalogram (EEG). Figure 1 shows an overview of the activities on a study day. All tests, with exception of the VVLT-15 and the FACE, were performed twice at baseline and repeatedly at 1 h, 2.5 h, and 3.5 h after drug administration on each study day. The VVLT-15 and the FACE were only performed once on each study visit.

For executive functioning, our main outcome was the change in performance on the adaptive tracker between baseline measurements and each time point after drug administration. For memory, our main outcome was the performance on the VVLT-15 after drug administration. Performance on the other NeuroCart® tests was the secondary outcome. We used alternate forms for tests performed repeatedly, and all tests were practiced on the screening day to prevent learning effects.

### Pharmacokinetic assessment

On each study day, venous blood samples were collected at 1 h, 2.5 h, and 3.5 h after administration (Fig. 1). We could not collect venous blood samples for two patients on all study visits, for one patient after methylphenidate administration, and for a different patient after galantamine administration. We could not collect venous blood samples at the first time point for one patient. For the analysis of galantamine and methylphenidate, two dedicated liquid chromatography-mass spectrometry methods/mass spectrometry were developed that were specific and sensitive for the analysis of interest. Bioanalysis was performed by the Pharmacy at the Amsterdam UMC.

### Safety assessments

On each study visit, safety measurements were performed, consisting of vital signs and a 12-lead ECG. All adverse events reported spontaneously by the patient or observed by the investigator or her staff were recorded on the adverse event data collection form using previously described protocol [19].

### Statistical analyses

All pharmacodynamic end points were summarized by drug and time. Statistical analyses of repeatedly measured variables were performed using mixed-model analysis of variance (ANOVA). We used the change in performance between the mean baseline measurements and each time point as dependent variable with treatment, period, time, and treatment by time as fixed factors using SAS for Windows V9.4 (SAS Institute, Inc., Cary, NC, USA). Participant, participant by treatment, and participant by time were included as random factors. The average baseline measurement was entered as covariate for each test on each time point. For all variables, estimated differences in mean change for each contrast with *p* value and 95% confidence interval were calculated. Single-measured parameters, as the VVLT-15 and FACE, without pre-value measurements were analyzed with a mixed-model ANOVA with treatment and period as fixed factors and participant as random factor. The general treatment effect and specific contrasts are reported with the estimated difference and the 95% confidence interval, the least square mean estimates, and the *p* value. All statistical hypothesis tests were conducted at alpha = 0.05 (two sided). No adjustments for multiple comparisons were applied.

As a preliminary exploration of the relationships between plasma concentrations of galantamine and methylphenidate and the performance on the VVLT-15 and the adaptive tracker, the data was plotted and correlated to evaluate the relationship graphically. The relationship was analyzed using a linear mixed effects model with intercept and slope as fixed effects and participant as random effect on intercept. The model was fitted using maximum likelihood with the lmer function in R.

## Results

### Patients

We included 30 patients with VCI; Table 1 shows the demographics and baseline characteristics. The study sample consisted of patients with a mean age of 67 ± 8 years, of whom 9 (30%) were female and 14 (47%) were clinically diagnosed with MCI. Small vessel disease (either white matter hyperintensities (Fazekas ≥ 2), lacunes or microbleeds, or a combination) was present in all patients, and in 7 patients, 1 or more cortical infarcts were also present.

**Table 1:** Demographics and baseline characteristics

	Total (30)
Demographics	
Age, mean (SD)	67 (8)
Females, *n* (%)	9 (30)
MMSE score, mean (SD)	26 (3)
CDR, median (IQR)	0.5 (0.5–1.0)
MCI, *n* (%)	14 (47)
Dementia, *n* (%)	16 (53)
Vascular risk factors	
Hypertension, *n* (%)	18 (60)
Hypercholesterolemia, *n* (%)	12 (40)
Diabetes mellitus, *n* (%)	4 (13.3)
Current Smoking, *n* (%)	5 (16.7)
MRI characteristics	
WMH (Fazekas), median (IQR)	2.5 (2–3)
Fazekas ≥ 2, *n* (%)	28 (93)
≥ 1 microbleed, *n* (%)	22 (73)
≥ 1 lacune, *n* (%)	13 (43)
Cortical infarct, *n* (%)	7 (23)
MTA, median (IQR)	1.5 (1–2)

*Abbreviations*: *CDR* Clinical Dementia Rating score, *IQR* interquartile range, *MCI* mild cognitive impairment, *MMSE* Mini-Mental State Examination, *MTA* medial temporal lobe atrophy, *WMH* white matter hyperintensities, *SD* standard deviation

Twenty-six patients completed the trial and received both study drugs and placebo. After the study visit with galantamine administration, 3 patients quit study participation due to side effects, namely anxiety feelings (*n* = 1), nausea and vomiting (*n* = 1), and bradycardia (*n* = 1). One patient was not able to come to the last study visit due to personal circumstances. Overall, 29 patients received galantamine administration, 28 patients received methylphenidate, and 27 patients received placebo.

### Pharmacodynamics

#### Main outcomes

**Fig. 2:**
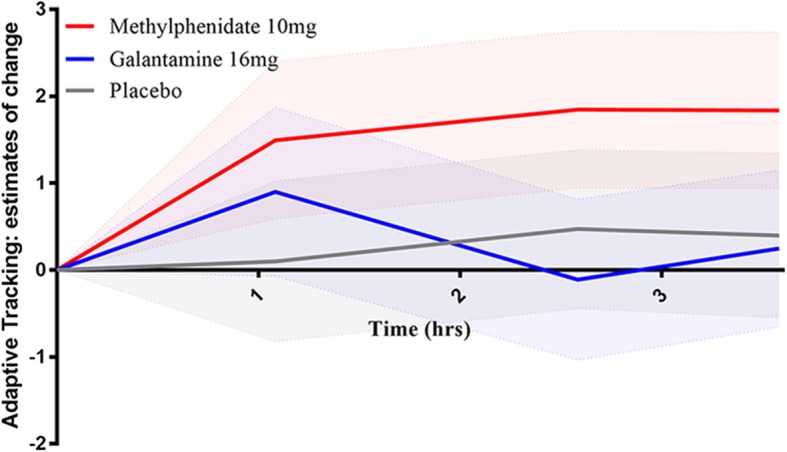
Effect of the study drugs on adaptive tracker. The shaded area represents the 95% CI. The estimated difference in mean change from baseline between methylphenidate and placebo was significant (*p* = 0.002)

Figure 2 shows the change in performance after drug administration on the adaptive tracker. The improvement in performance on the adaptive tracker after methylphenidate administration was higher than after placebo (estimated difference in mean performance (%) compared to baseline, 1.40% (95% confidence interval [CI] 0.56–2.25), *p* = 0.002). We saw no effect of galantamine on the adaptive tracker. Table 2 gives the results of the study drugs on memory function. Patients scored better on the third trial of immediate word recall of the VVLT-15 after a single dose of methylphenidate. Patients remembered fewer words after galantamine administration on delayed word recall than after placebo.

**Table 2:** Pharmacodynamic outcome: memory

Parameter	Galantamine—placebo	Methylphenidate—placebo
Word recall correct 1	− 0.06 (− 0.79, 0.68)	0.23 (− 0.50, 0.96)
Word recall correct 2	− 0.24 (− 0.91, 0.42)	0.48 (− 0.17, 1.13)
Word recall correct 3	− 0.52 (− 1.09, 0.04)	0.59 (0.03, 1.15)*
Delayed word recall correct	− 0.84 (− 1.65, − 0.03)*	− 0.05 (− 0.84, 0.74)
Delayed word recognition correct	− 1.01 (− 2.08, 0.06)	− 0.20 (− 1.23, 0.83)

Numbers are the difference in remembered words between two study drugs with 95% CI

*p < 0.05

#### Secondary outcomes

Additional file 1: Table S1 gives the results of the study drugs on all outcomes. We found no effect of methylphenidate on the N-back task or the Stop Signal Task. After methylphenidate administration, patients did not remember more faces in the FACE test, albeit we did find a faster reaction time on recognizing a familiar face on the FACE test compared to placebo (estimates of difference − 178.5 ms (95% CI − 329.2, − 27.8; *p* = 0.02)). After administration of methylphenidate, the change in saccadic peak velocity (estimated difference in change 35.03 (12.04, 58.02; *p* < 0.004)) was higher compared to placebo. We found no effect of methylphenidate on the VAS scales.

Administration of galantamine had a positive effect on the mean reaction time of the Stop Signal Task on the Go Trials compared to placebo (estimated difference in change from baseline − 51.40 ms (− 95.90, − 6.89; *p* = 0.02)). After administration of galantamine, patients were less inaccurate on the saccadic pursuit compared to placebo (estimated difference in mean change from baseline − 1.17 (− 1.92, − 0.42; *p* = 0.003)). Furthermore, we observed a reduction in delta power over the Fz-Cz and Pz-Oz leads (estimated difference in mean change from baseline − 14.1% (− 23.8, − 3.1%; *p* = 0.01) and − 13.1 (− 23.8, − 0.8%; *p* = 0.04)) compared to placebo. We found overall effects of galantamine on the VAS scales for mood (estimated difference in mean change from baseline − 4.79 (− 9.22, − 0.37; *p* = 0.03)) and for external (0.11 (0.03, 0.19; *p* = 0.01)) and internal perception (0.09 (0.05, 0.14; *p* = 0.0003)). We found no effects of galantamine on the N-back or the FACE test compared to placebo.

### Pharmacokinetics

Concentrations of methylphenidate and galantamine were determined in plasma. An overview of the individual plasma concentrations of methylphenidate and galantamine on the three time points after drug administration is provided in Additional file 1: Figure S1. The slope of the linear relationship between methylphenidate and adaptive tracker was estimated to be 0.162 percentage point per nanogram per milliliter (standard error 0.105; *p* = 0.13). Although the slope was not significant, it may be indicative of an increased effect of adaptive tracking with increasing dose. No indication of an exposure-response relationship existed for methylphenidate and VVLT-15 or for galantamine with adaptive tracker or VVLT-15.

### Safety

No serious adverse events or other significant adverse events occurred. The most common adverse events after methylphenidate administration were hypervigilance in 4/28 (14%) patients, headache in 3 (11%) patients, and dizziness in 2 (7%) patients, which are all known as frequent side effects of methylphenidate. The most frequent adverse event seen in patients after galantamine administration was nausea in 23/29 (79%) patients. Thirteen of these patients also vomited. When comparing the concentrations of patients who vomited to patients who did not vomit, we found no differences in mean concentration galantamine for each time point. Other frequent side effects were dizziness in 11 (38%) patients, fatigue in 7 (24%) patients, diarrhea and hyperhidrosis in 4 patients (14%), and abdominal discomfort/pain in 3 (10%) patients. All adverse events were self-limiting, and most were mild or moderate in intensity, except for 1 patient with severe dizziness and 2 patients with severe vomiting after galantamine. Table 3 shows the mean changes in blood pressure and heart rate after drug administration. We measured the highest increase in blood pressure in patients after galantamine administration. Bradycardia (heart rate < 50/min) occurred in 3 patients (heart rate = 48/min, heart rate = 47/min, and heart rate = 36/min) after galantamine administration.

**Table 3:** Mean change in blood pressure and heart rate

Study medication	Mean change of systolic blood pressure	Mean change of diastolic blood pressure	Mean change of heart rate
Methylphenidate	3.3 ± 9.2	0.5 ± 6.3	− 0.5 ± 6.0
Galantamine	12.6 ± 15.4	5.6 ± 8.6	− 5.1 ± 6.7
Placebo	0.6 ± 7.6	1.5 ± 6.0	− 2.5 ± 5.9

Numbers are in mm Hg for blood pressure and heart rate is in beats/min with standard deviation

## Discussion

In the STREAM-VCI study, a single dose of methylphenidate improved executive functioning and immediate recall in patients with VCI, whereas a single dose of galantamine did not. These functions were predefined as the main outcomes of the study. On secondary endpoints, both drugs caused small accelerations of CNS activity, albeit on different parameters. Methylphenidate was well tolerated. Galantamine produced gastrointestinal side effects in a considerable number of patients, which might have influenced the results.

To date, no symptomatic treatment for patients with VCI is available. Evidence for a beneficial effect of methylphenidate in patients with VCI is sparse [12]. The results of the STREAM-VCI study show that methylphenidate may be an effective symptomatic treatment for VCI patients. Methylphenidate has also been shown to have a small effect on executive functioning and memory in healthy individuals [21, 22]. Effects of methylphenidate on cognition in healthy adults have been shown to be small, highly variable, and baseline dependent, which may suggest that only patients with low performance benefit from the methylphenidate [23, 24]. There is some evidence that in healthy controls, low sustained attention is associated with reduced dopamine receptor availability in the left caudate and that methylphenidate can improve performance by elevating dopamine levels [24]. This might implicate that only in subjects with deficits in the monoaminergic system, methylphenidate can enhance cognition.

In patients with AD, methylphenidate has shown to be able to reduce apathy [25]. Apathy is very common in VCI and is associated with executive dysfunction/processing speed [26]. Apathy can be measured with several questionnaires such as the Apathy Evaluation Scale (AES) [27], the neuropsychiatric inventory [28], and the geriatric depression score [29]. In the present study, we did not use questionnaires to measure apathy and therefore have no insight on the presence and severity of the apathy syndrome in our study sample. As apathy is frequently seen in VCI, we do believe that some patients might have had apathy. We cannot rule out that methylphenidate might have caused an improvement in executive function by reducing associated apathy.

After administration of methylphenidate, an increase in gamma signal on EEG was seen compared to placebo. The gamma signal has often been neglected in previous studies, as there are technical measurement difficulties. More recent studies investigating gamma frequencies suggest a relation with cognitive functioning, especially working memory and attention. These studies have shown that drugs influencing the dopamine activity, such as methylphenidate, influence gamma power [30]. Thus, the effect of methylphenidate seen in this study on executive functioning and gamma power corresponds with the literature stating that gamma power is associated with working memory and attention. This assumption must be taken with caution, as gamma power is also known to be caused by increased scalp muscle activity, which can also be an effect of methylphenidate [31]. More studies are necessary to investigate the effect of methylphenidate on gamma power and the relationships between gamma power and cognition.

In the present study, methylphenidate was well tolerated after a single dose. In previous trials, side effects were reported in patients when methylphenidate was taken for a longer period of time [12, 32]. Most important side effects are an increase in heart rate and blood pressure, which led to reluctance in subscribing methylphenidate to patients with cardiovascular disease. However, in a population-based cohort of young and middle-aged adults, the use of ADHD medication, including methylphenidate, was not associated with an increased risk of cardiovascular events [33]. In trials investigating the use of methylphenidate in older patients with AD (mean age > 75), side effects were modest. However, symptomatic cardiovascular disease was an exclusion criterion in these trials [8, 9]. Follow-up studies with cardiovascular monitoring are necessary to investigate whether repeated/prolonged methylphenidate administration is safe in older patients with cardiovascular disease before implemented in a clinical practice.

Galantamine has been shown to be effective in patients with AD [14]. The effects in VCI, however, have been inconsistent [15]. In the STREAM-VCI study, we found no positive effect of galantamine on memory or executive functioning. Comorbid AD could be related to treatment responsiveness of galantamine. Results for galantamine did not change when data was re-analyzed with patients with VCI alone (data not shown).

Previous studies have shown that acetylcholine is important not only for memory function, but also in the attentional “search” processes which might explain the acceleration of the reaction time of the Go Trials of the Stop Signal Task, and some improvement of saccadic inaccuracy on visually guided saccadic eye movements [34]. Furthermore, galantamine showed an (trend to) acceleration of EEG activity, by a reduction of delta (*p* = 0.01) and theta signal power (*p* = 0.06). While slowing of EEG has been recognized in patients with dementia, and has been used as a marker for interventions in clinical trials in AD, the effect of cholinesterase inhibitors on EEG is not consistent. There is some evidence that cholinesterase inhibitors may reduce theta and delta power, but these studies were small, relatively old, and not designed according to the current standards [35, 36]. Overall, the neurophysiological alterations after a single dose of galantamine could implicate that galantamine may enhance CNS activity, which in at least some VCI patients might facilitate cognitive improvements during prolonged treatment. To assess subtle drug effects (mood, internal and external effects), we used the VAS Bond and Lader; however, this test is difficult to understand for this patient population and results are difficult to interpret.

The high number of cholinergic side effects of galantamine may be one explanation for a decreased performance on some of the more demanding tasks, such as the memory tests, which showed no improvement and even a small decline in delayed recall memory. The amount and severity of side effects seen are consistent with a recent trial [37]. An explanation could be that patients were given 16 mg galantamine without an up-titration period, as is done in clinical practice according to the label. Based on an ongoing trial (CHDR0915), we assumed that galantamine would only be effective in a relatively high dose and that this would be tolerated well enough to be given in this dose. Up-titration of galantamine may improve tolerability and thus efficacy in patients with VCI. Future studies will need to examine the responses in patients with VCI after up-titration of galantamine, at least to dose levels that have proven to be efficient in AD [38]. Prolonged stimulation of cholinergic CNS processes may also lead to clinical improvements in VCI, as in AD increased improvements are seen after several months of treatment [39].

A strength of the STREAM-VCI study is the crossover design, where every patient is its own control, requiring less patients to find an effect. A disadvantage of this design is that carry-over effects may influence results; hence, we included a washout period of 1 week. Another strength of this study is that we used a standardized CNS test battery. The NeuroCart® uses repeated measurements of relatively simple tests and is able to reliably measure limited drug effects in small groups [20]. The simplicity of the tests reduces the influence of factors like motivation, comprehension, boredom or, like in this study, physical discomfort caused by side effects. This may have been part of reason why the limited effects of galantamine were only shown by some NeuroCart® tests. Moreover, relatively simple CNS tests avoid some of the problems associated with cognitive tests, which almost by definition always involve different collaborating CNS functions, making it challenging to deduct which mechanism is affected by a cognition enhancing compound.

Trials investigating symptomatic treatment in VCI are urgently needed, as no treatment is available for this patient group [1]. Patients with VCI represent a heterogenic patient population, with a variety of vascular brain injury (e.g., white matter hyperintensities, strategic and/or a large cortical infarcts) causing cognitive impairment [1]. Different distributions of vascular lesions across the brain may result in impairments of different neurotransmitter systems in different VCI patients. This may have consequences for treatment selection, in that one patient might benefit from a cholinergic treatment and another from a monoaminergic treatment. Heterogeneity of affected neuropharmacological systems in VCI may also have caused variability of the effects of galantamine and methylphenidate in our study.

## Conclusion

The proof-of-principle study STREAM-VCI shows that methylphenidate may be a candidate for symptomatic treatment of patients with VCI. Future studies should investigate the efficacy and safety of prolonged administration of methylphenidate in VCI patients. Galantamine showed no benefical effect on the main outcome. The effects of galantamine in VCI should be investigated with an up-titration period.

## Supplementary information

**Additional file 1: Table S1.** Pharmacodynamic Outcomes. **Figure S1.** Overview of the concentration of methylphenidate (A) and galantamine (B) with colors representing adverse events.

## Data Availability

All data analyzed during this study are included in this published article (and its additional files). Anonymized raw data may be shared upon request from any qualified investigator provided that data transfer is in agreement with European Union legislation on the general data protection regulation.

## Abbreviations

AD: Alzheimer’s disease
ANOVA: Analysis of variance
CDR: Clinical Dementia Rating score
CI: Confidence interval
CNS: Central nervous system
FACE: Face Encoding Recognition Task
EEG: Electroencephalogram
MCI: Mild cognitive impairment
MMSE: Mini-Mental State Examination
MRI: Magnetic resonance imaging
VAS: Visual Analog Scale
VCI: Vascular cognitive impairment
VVLT-15: Visual Verbal Learning Test-15
