# Supplementary material for: Methylphenidate and galantamine in patients with vascular cognitive impairment–the proof-of-principle study STREAM-VCI
Source: Alzheimers Res Ther. 2020 Jan 7;12:10. doi: 10.1186/s13195-019-0567-z (PMC6947990; doi:10.1186/s13195-019-0567-z)
Supplement: Supplementary file 1 — Additional file 1: Table S1. Pharmacodynamic Outcomes. Figure S1. Overview of the concentration of methylphenidate (A) and galantamine (B) with colors representing adverse events. [file 13195_2019_567_MOESM1_ESM.pdf]

Additional File 1

Supplementary Table 1: Pharmacodynamic Outcomes

Parameter	Treatment p-value	Galantamine Placebo	Methylphenidate Placebo
**Memory**			
VVLT-15: Word recall correct 1	0.71	-0.06 (-0.79, 0.68) p=0.88	0.23 (-0.50, 0.96) p=0.54
VVLT-15: Word recall correct 2	0.09	-0.24 (-0.91, 0.42) p=0.46	0.48 (-0.17, 1.13) p=0.15
VVLT-15: Word recall correct 3	>0.001	-0.52 (-1.09, 0.04) p=0.07	0.59 (0.03, 1.15) p=0.04*
VVLT-15: Delayed word recall correct	0.07	-0.84 (-1.65, -0.03) p=0.04*	-0.05 (-0.84, 0.74) p=0.90
VVLT-15: Delayed word recognition correct	0.15	-1.01 (-2.08, 0.06) p=0.06	-0.20 (-1.23, 0.83) p=0.70
FACE: number correct	0.72	0.52 (-0.84, 1.88) p=0.45	0.09 (-1.22, 1.40) p=0.89
**Executive functioning**			
Adaptive tracking (%)	0.002	0.02 (-0.84, 0.88) p=0.96	1.40 (0.56, 2.25) p=0.002
N-back correct 0	0.91	-0.03 (-0.17,0.11) p=0.67	-0.01 (-0.15,0.12) p=0.85
N-back correct 1	0.49	0.04 (-0.25,0.32) p=0.79	0.15 (-0.12,0.42) p=0.26
N-back correct 2	0.56	-0.17 (-0.63, 0.29) p=0.46	-0.23 (-0.66, 0.21) p=0.29
N-back mean RT 0 back (ms)	0.96	-1.5 (-29.0, 25.9) p=0.91	-3.8 (-30.2, 22.5) p=0.77
N-back mean RT 1 back (ms)	0.40	-12.2 (-56.1, 31.7) p=0.58	-28.2 (-70.2, 13.9) p=0.18
N-back mean RT 2 back (ms)	0.60	17.9 (-50.6, 86.4) p=0.60	-15.8 (-80.0, 48.3) p=0.62
SST: Total correct Go-trials	0.23	7.33 (-1.57, 16.24) p=0.10	5.42 (-3.22, 14.05) p=0.21
SST: Total missed Go-trials	0.48	-5.70 (-15.34, 3.93) p=0.24	-3.69 (-13.07, 5.69) p=0.43
SST: Mean RT Go-trials (ms)	0.06	-51.40 (-95.90, -6.89) p=0.02*	-9.89 (-53.11, 33.33) p=0.65

SST: Stop Signal RT (ms)	0.39	-3.37 (-44.80, 38.06) p=0.87	-25.49 (-66.00, 15.02) p=0.21
SST: Total correct Stop-trials	0.42	-0.56 (-2.53, 1.42) p=0.58	0.73 (-1.18, 2.63) p=0.45
SST: Mean RT Stop-trials (ms)	0.58	-31.03 (-90.44, 28.38) p=0.30	-16.19 (-74.41, 42.04) p=0.59
SST: Mean SSD Stop-trials	0.12	-43.32 (-102.73, 16.09) p=0.15	16.12 (-41.37, 73.61) p=0.58
**Other Tests**			
FACE: mean RT correct (ms)	0.06	-117.0 (-274.3, 40.2) p=0.14	-178.5 (-329.2, -27.8) p=0.02*
Smooth Pursuit (%)	0.06	1.58 (-0.44, 3.60) p=0.12	-0.86 (-2.76, 1.05) p=0.37
Saccadic Inaccuracy (%)	0.01	-1.17 (-1.92, -0.42) p=0.003**	-0.60 (-1.30, 0.09) p=0.09
Saccadic Peak Velocity (deg/s)	0.001	-7.41 (-31.16, 16.33) p=0.53	35.03 (12.04, 58.02) p=0.004**
Saccadic Reaction Time (sec)	0.57	-0.002 (-0.013, 0.009) p=0.72	-0.005 (-0.016, 0.005) p=0.30
VAS Alertness (mm)	0.02	-4.50 (-9.99, 0.98) p=0.10	3.36 (-1.96, 8.67) p=0.21
VAS Calmness (mm)	0.23	-1.14 (-6.66, 4.39) p=0.68	-4.34 (-9.66, 0.97) p=0.11
VAS Mood (mm)	0.10	-4.79 (-9.22, -0.37) p=0.03*	-2.29 (-6.58, 1.99) p=0.28
VAS External log(mm)	0.005	0.11 (0.03, 0.19) p=0.01*	-0.02 (-0.01, 0.05) p=0.57
VAS Internal log(mm)	0.0003	0.09 (0.05, 0.14) p=0.0003**	0.004 (-0.04, 0.05) p=0.87
VAS feeling high log(mm)	0.39	0.08 (-0.05, 0.21) p=0.21	0.01 (-0.11, 0.14) p=0.86
EEG Alpha Fz-Cz (uV)	0.05	-4.7% (-16.4%, 8.6%) p=0.46	11.9% (-1.8%, 27.4%) p=0.09
EEG Alpha Pz-Oz (uV)	0.05	-4.9% (-17.1%, 9.1%) p=0.47	12.4% (-2.0%, 28.9%) p=0.09
EEG Beta Fz-Cz (uV)	0.03	-4.6% (-13.5%, 5.3%) p=0.34	9.3% (-0.9%, 20.4%) p=0.07
EEG Beta Pz-Oz (uV)	0.08	-1.4% (-12.5%, 11.1%) p=0.81	11.7% (-0.6%, 25.6%) p=0.06
EEG Delta Fz-Cz (uV)	0.002	-14.1% (-23.8%, -3.1%) p=0.01*	7.7% (-4.4%, 21.2%) p=0.22
EEG Delta Pz-Oz (uV)	>0.05	-13.1% (-23.8%, -0.8%) p=0.04*	1.0% (-11.3%, 14.9%) p=0.88

EEG Gamma Fz-Cz (uV)	0.01	-1.8% (-11.6%, 9.1%) p=0.73	13.8% (2.6%, 26.3%) p=0.02*
EEG Gamma Pz-Oz (uV)	0.11	-1.1% (-13.9%, 13.6%) p=0.88	12.9% (-1.5%, 29.4%) p=0.08
EEG Theta Fz-Cz (uV)	0.03	-10.4% (-20.2%, 0.6%) p=0.06	5.0% (-6.3%, 17.7%) p=0.39
EEG Theta Pz-Oz (uV)	0.55	-6.4% (-18.0%, 6.9%) p=0.32	-0.7% (-12.9%,13.2%) p=0.92

Numbers are the difference between two study drugs with 95%CI. *p<0.05, **p<0.01 difference. EEG = electroencephalogram, FACE = Face Encoding Recognition Task, RT = reaction time, SST = Stop Signal Task, VAS = Visual Analog Scale, VVLT-15 = Visual Verbal Learning Test-15.

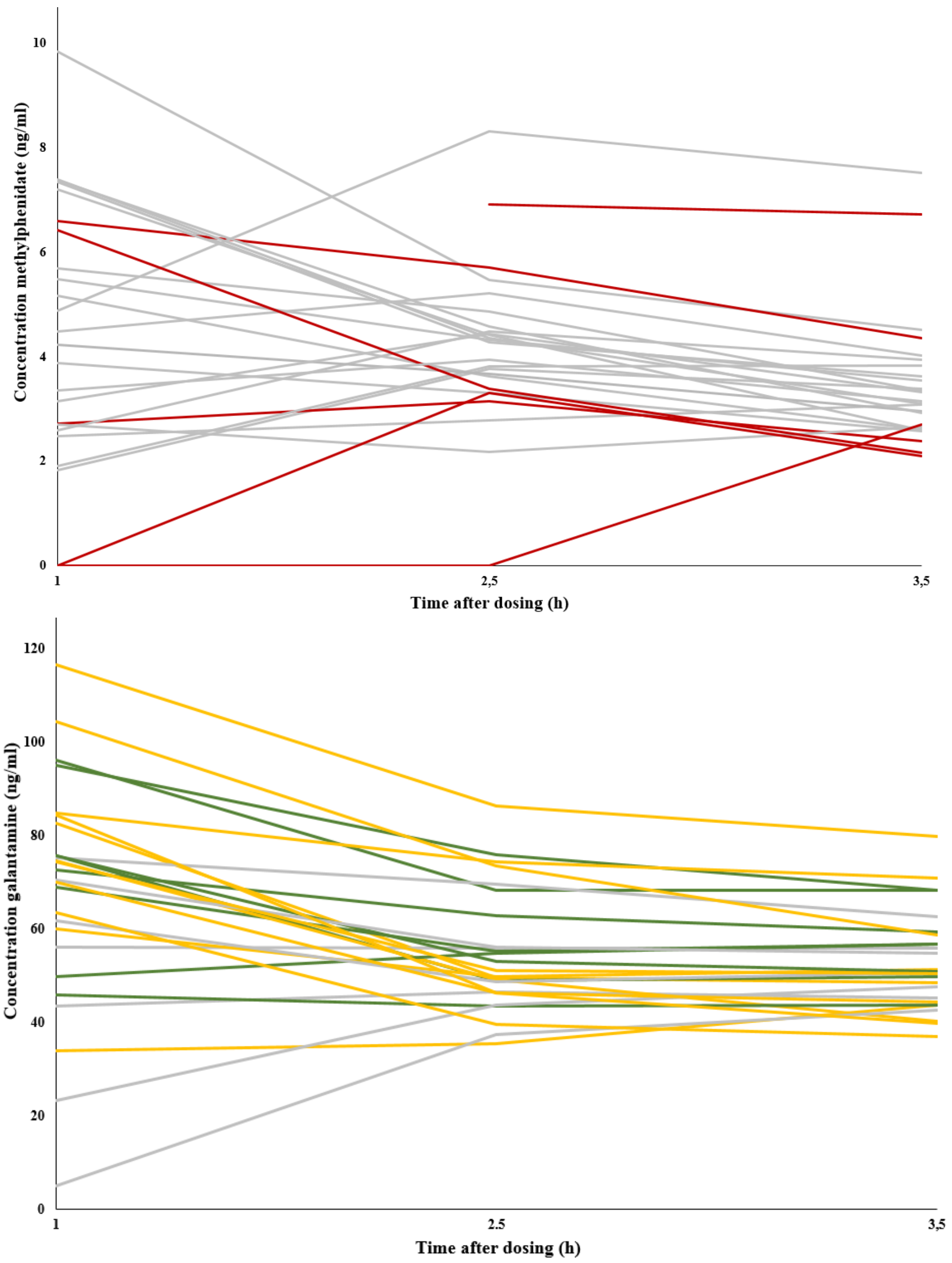

Supplementary Figure 1. Overview of the concentration of methylphenidate **(A)** and galantamine **(B)** with colors representing adverse events. Each line represents one patient. (**A**) The red lines are patients with a significantly increased blood pressure, lines in grey are patients without significantly increased blood pressure. (**B**) Orange lines represent patients who vomited, green lines patients who experienced nausea, and grey lines are patients with no symptoms or other symptoms than nausea or vomiting.
